# *Clinacanthus nutans* Leaves Extract Reverts Endothelial Dysfunction in Type 2 Diabetes Rats by Improving Protein Expression of eNOS

**DOI:** 10.1155/2020/7572892

**Published:** 2020-08-14

**Authors:** Ahmad Khusairi Azemi, Siti Safiah Mokhtar, Aida Hanum Ghulam Rasool

**Affiliations:** ^1^Department of Pharmacology, School of Medical Sciences, Universiti Sains Malaysia, 16150 Kota Bharu, Kelantan, Malaysia; ^2^Hospital Universiti Sains Malaysia, Kubang Kerian, 16150 Kota Bharu, Kelantan, Malaysia

## Abstract

Diabetes mellitus is associated with endothelial dysfunction; it causes progressive vascular damage resulting from an impaired endothelium-dependent vasorelaxation. In the diabetes state, presence of hyperglycemia and insulin resistance predisposes to endothelial dysfunction. *Clinacanthus nutans*, widely used as a traditional medicine for diabetes is reported to have hypoglycemic, hypolipidemic, antioxidant, and anti-inflammatory properties. However, the possibility of *C. nutans* affecting the vascular endothelial function in diabetes remains unclear. This study was aimed at evaluating the effects of *C. nutans* methanolic leaves extract (CNME) on endothelial function in a type 2 diabetes (T2DM) rat model. Sixty male Sprague-Dawley rats were divided into five groups (*n* = 12 per group): nondiabetic control, nondiabetic treated with four weeks of CNME (500 mg/kg/daily), untreated diabetic rats, diabetic treated with metformin (300 mg/kg/daily), and diabetic treated with CNME (500 mg/kg/daily). T2DM was induced by a single intraperitoneal injection of low-dose streptozotocin (STZ) to rats fed with high-fat diet (HFD). Endothelial-dependent and endothelial-independent relaxations and contractions of the thoracic aorta were determined using the organ bath. Aortic endothelial nitric oxide synthase (eNOS) expression was determined using Western blotting. Endothelial-dependent relaxation was reduced in diabetic rats. Both diabetic groups treated with CNME or metformin significantly improved the impairment in endothelium-dependent vasorelaxation; this was associated with increased expression of aortic eNOS protein. CNME- and metformin-treated groups also reduced aortic endothelium-dependent and aortic endothelium-independent contractions in diabetics. Both of these diabetic-treated groups also reduced blood glucose levels and increased body weight compared to the untreated diabetic group. In conclusion, *C. nutans* improves endothelial-dependent vasodilatation and reduces endothelial-dependent contraction, thus ameliorating endothelial dysfunction in diabetic rats. This may occur due to its effect on increasing eNOS protein expression.

## 1. Introduction

Cardiovascular disease is the leading cause of death worldwide. Diabetes mellitus is an important independent risk factor for the development of atherosclerosis and cardiovascular diseases. Endothelial cells line the inner blood vessels; it regulates vascular tone and maintains the vascular homeostasis. Dysfunction of the endothelium, termed endothelial dysfunction (ED), refers to diminished availability of the primary endothelium-derived relaxing factor (EDRF) nitric oxide (NO) and increased production of vasoconstrictors [1]. ED plays a central role in the pathogenesis of atherosclerosis in diabetes and other cardiovascular diseases. It occurs early, before morphological changes are visible in the vessel wall. NO is formed in the endothelial cells via enzymatic action of endothelial NO synthase (eNOS) with several cofactors such as tetrahydrobiopterin and nicotinamide adenine dinucleotide phosphate (NADPH).

*Clinacanthus nutans* (Burm. f.) Lindau, commonly called Sabah Snake Grass or “*Belalai Gajah*,” is widely used in Malaysia, Indonesia, and Thailand as a traditional medicine for diabetes and inflammation and other medical conditions [2, 3]. Reports have emerged on the antioxidant, antidiabetic, hypolipidemic, and anti-inflammatory properties of *C. nutans* extracts. These are properties that are generally considered useful to delay or prevent atherosclerosis in diabetes. In the diabetes state, exposure of arterial tissue to high glucose and free fatty acid concentration induces superoxide production and increased oxidative stress. Superoxide anion reacts with NO to form peroxynitrite which eliminates the biological activity of NO. Superoxide anion also alters the catalytic activity of eNOS in endothelial cells impairing NO production. These reduce NO bioavailability in the vascular wall contributing to ED. Increased production of ROS may also affect the reduction of tetrahydrobiopterin, which uncoupled eNOS and promote production of superoxide, rather than NO [1]. Sarega et al. showed that the extract of *C. nutans* increased serum superoxide dismutase (SOD) enzyme and catalase levels and reduced lipid peroxidation [4]. Free radical species such as superoxide anions can be neutralized by endogenous antioxidants such as SOD and catalase [5].

*C. nutans* extract has been reported to inhibit *α*-glucosidase enzyme activity [6, 7]. The inhibition of this enzyme reduces intestinal carbohydrate metabolism by retarding the cleavage of polysaccharide to glucose; this will reduce glucose intestinal absorption [7]. This effect was supported by a recent study that demonstrated lowering of blood glucose concentrations in a type 2 diabetic (T2DM) rat model treated with *C. nutans* [8]. As mentioned above, chronic hyperglycemia can impair NO bioavailability causing ED. Thus, the hypoglycemic property of *C. nutans* may also ameliorate ED.

Hyperlipidemia increases ROS production, resulting in oxidation and peroxidation of lipids, protein, and lipoprotein [9]. Peroxidation of endothelial cell membrane can lead to endothelial damage and dysfunction. Low-density lipoprotein cholesterol (LDL-C) and especially its oxidized form (ox-LDL-C) play a pivotal role in ED and atherogenesis [10, 11]. Ox-LDL-C increases synthesis of caveolin-1, which inactivates eNOS [12]; ox-LDL-C also reduces the expression of eNOS protein, resulting in impairment of NO-mediated vasodilatory response [13, 14]. *C. nutans* extracts have been reported to reduce serum LDL-C levels in high-fat diet- (HFD-) induced T2DM rat model [8], and the hypercholesterolemic rat model [4].

Inflammation is a significant contributor to ED at the arterial intima layer; monocytes release a number of inflammatory cytokines such as interkeukin-1 (IL-1), IL-6, and tumor necrosis factor-alpha (TNF-*α*), all of which may contribute to vascular endothelium injury [15]. TNF-*α* activates NADPH oxidase, which augments superoxide generation leading to a reduction in NO bioavailability [16]. TNF-*α* also affects the half-life of eNOS mRNA further affecting NO production [17]. Mai et al. had reported that the extract of *C. nutans* reduced the levels of inflammatory cytokines which include TNF-*α* [18].

Currently, the effect of *C. nutans* extract on ED in diabetes is not known. T2DM is the predominant diabetes type in humans. Thus, this study is aimed at evaluating the effects of CNME on endothelial function in a T2DM rat model. T2DM was induced in rats fed a high-fat diet (HFD) and given low-dose STZ injection, which is analogous to development of T2DM in humans [19]. Endothelium-dependent and endothelium-independent relaxations and contractions of the aorta were studied. Aortic eNOS protein expression was also quantified. Outcome parameters were compared to metformin, a biguanide derivative widely used in the management of T2DM.

## 2. Materials and Methods

### 2.1. Plant Material and Preparation of the Extract

Fresh leaves of *C. nutans* were obtained from their natural habitat in Pasir Puteh, a district in the state of Kelantan, Malaysia. The plant species was authenticated by a botanist from Forest Research Institute Malaysia (FRIM), Kepong, Selangor. A voucher specimen, SBID 039/18, has been deposited at the herbarium of Natural Product Division FRIM. leaves extraction was carried out according to the method previously described by Abdul Rahim et al. [20]. *C. nutans* leaves were dried in an oven at 40°C for 3 days and ground into powder form using an electric grinder. To obtain CNME, 125 g of *C. nutans* leaves powder was soaked in methanol (Fisher Scientific, Loughborough, England) in the ratio of 1 : 20 (*w*/*v*) for 72 hours at room temperature. The mixture was filtered using a filter funnel and Whatman No. 1 (Merck, Darmstadt, Germany) filter paper. The residue of the filtration was weighted again and soaked with methanol within the same ratio. The filtration and soaking processes were repeated twice on the residue. The filtrate collected from each extraction was pooled and evaporated in vacuum rotary evaporator (Heidolph, Germany) at 40°C under reduced pressure to obtain CNME extract. This extraction yielded approximately 38.91 g of dried crude CNME (yield was 31.13% (*w*/*w*)), which was then stored at 4°C until it was used.

### 2.2. Gas Chromatography-Mass Spectrometry (GC-MS) Analysis of CNME

GC-MS analysis was carried out to identify the chemical constituents in the CNME extract. The method described by Abdul Rahim et al. [20] was adapted to carry out the GC-MS analysis of CNME using Agilent 7890A (Agilent Technologies, USA) gas chromatography coupled with MSD quadrupole detector 5975C (Agilent Technologies).

### 2.3. Animals and Experimental Protocols

The experimental protocol used in this study was approved by the Universiti Sains Malaysia (USM) Institutional Animal Care and Use Committee (USM IACUC) (USM/IACUC/2017/(105)(834)). Male Sprague-Dawley rats (12 weeks old) weighing between 250 and 300 g were divided into nondiabetic and diabetic groups. The nondiabetic group was fed with a standard commercial food pellet (Gold Coin Feedmills, Port Klang, Malaysia), while the diabetic group was fed with HFD throughout the study period. The HFD was prepared according to the methods described by Lim et al. [21] and Ishak et al. [22] with modifications. It was prepared from a mixture of 50% commercial food pellet, 38% ghee, 8% full cream milk powder, and 4% white sugar. Rats in the diabetic group were fed with HFD for 4 weeks, before injected intraperitoneally (i.p.) with a single low-dose STZ (40 mg/kg, dissolved in 10 mM citrate buffer, 1 mL/kg). Rats in the nondiabetic group were injected with an equal volume of citrate buffer (1 mL/kg, i.p.) [22, 23]. A week later, fasting blood glucose (FBG) concentrations were measured from the blood in the tail vein using the one-touch glucometer (Accu-Check, Roche Diagnostic, Indianapolis, IN, USA). Rats with FBG concentrations ≥ 16.7 mmol/L were considered diabetic. After induction of diabetes, diabetic and nondiabetic rats continued with their respective diets until the eleventh week. At week 11, rats were divided into five groups of 12 rats each: nondiabetic control rats (C), nondiabetic rats treated with 500 mg/kg/daily of CNME extract (C+CNME), untreated diabetic rats (DM), diabetic rats treated with 300 mg/kg/daily of metformin (DM+Met), and diabetic rats treated with 500 mg/kg/daily of CNME extract (DM+CNME). Metformin and CNME were given via oral gavage to all treated rats for 4 weeks. Rats were then euthanized by intraperitoneal injection using a combination of ketamine (300 mg/kg) and xylazine (30 mg/kg).

### 2.4. Functional Study

The thoracic aorta was isolated from the rats and immediately placed in ice-cold and oxygenated physiological saline solution containing 118 mM sodium chloride (NaCl), 4.7 mM potassium chloride (KCl), 2.0 mM calcium chloride dehydrate (CaCl_2_·2H_2_O), 1.18 mM magnesium sulphate heptahydrate (MgSO_4_·7H_2_O), 25 mM sodium hydrogen carbonate (NaHCO_3_), 1.2 mM potassium dihydrogen phosphate (KH_2_PO_4_), and 5.5 mM D-glucose. The thoracic aorta was cleared of fat and cut into several rings (3 mm in length). In some preparations, the endothelial cell layers were removed by scrubbing the lumen of the rings with a blunt forceps. The rings were suspended between two stainless steel hooks in a 20 mL organ bath filled with physiological saline solution [24]. The bathing solution was continuously bubbled with carbogen (95% O_2_ and 5% CO_2_) and maintained at 37°C (pH 7.4). All rings were initially stretched to an optimal resting tension of 1.0 g and allowed to equilibrate for 60 min. Afterwards, the rings were contracted with KCl (60 mM) to obtain a reference contraction and to ensure smooth muscle viability. Then, the rings were rinsed with physiological saline solution and precontracted with phenylephrine (PE) (10^−6^ M). When a steady contraction to phenylephrine was obtained, acetylcholine (ACh) (10^−6^ M) was added to assess for the presence (or absence) of functional endothelial cells. To study endothelium-dependent relaxation, aortic rings were precontracted with PE (10^−6^ M) and exposed to cumulative concentrations of ACh (10^−9^-10^−4^ M). Cumulative concentrations of sodium nitroprusside (SNP) (10^−9^-10^−4^ M) were used as an endothelium-independent agonist. Exposing the aortic rings to cumulative concentrations of calcium ionophore A23187 (10^−9^-10^−5^ M), after 30-minute incubation with L-Nitro-Arginine Methyl Ester (L-NAME) (10^−4^ M), the NO synthase inhibitor assessed endothelium-dependent contraction. Cumulative concentrations of PE (10^−9^ -10^−4^ M) were used as an endothelium-independent agonist [23].

### 2.5. Western Blotting

Western blotting was performed according to the methods described by a previous study [23]. The thoracic aorta was dissected and homogenized in the lysis buffer (RIPA buffer, Sigma Chemical Co., St. Louis, MO, USA) with a protease inhibitor cocktail 0.05% (Sigma Chemical Co., St. Louis, MO, USA). Samples were centrifuged at 3,000 × g for 20 minutes at 4°C, and the supernatants were collected. Protein concentrations were determined using the protein determination kit (Cayman Chemicals, USA). In all immunoblot experiments, 30 *μ*g of proteins was loaded in each lane of 10% Sodium Dodecyl Sulphate- (SDS-) polyacrylamide gel. After electrophoresis, proteins were transferred to polyvinylidene difluoride (PVDF) membranes (Millipore Corp., Billerica, MA, USA) and incubated with primary antibodies against eNOS (1 : 1000; Cell Signaling, Danvers MA) and *β*-actin (1 : 1000; Cell Signaling, Danvers MA). After washing, the membranes were incubated with horseradish peroxidase- (HRP-) conjugated polyclonal secondary antibody (1 : 1000; Cell Signaling, Danvers MA) in a blocking buffer. All proteins were detected using Signal Fire ECL reagent (Cell Signaling, Danvers MA) with Fusion FX imaging systems and quantified using the ImageJ software. The relative protein presence of eNOS was calculated based on the ratio of the intensity of eNOS bands to the corresponding *β*-actin.

### 2.6. Statistical Analysis

Statistical analysis was performed using GraphPad Prism version 7.0 (San Diego, CA, USA). Relaxation was expressed as a percentage relative to the tension generated by 10^−6^ M of PE. Comparisons between the groups were made based on maximal responsiveness (*E*_max_) to the agonist studied. Results were presented as mean ± standard error of mean (SEM). Comparison of body weight was made using a two-way analysis of variance (ANOVA). Differences between groups for another parameter were compared using one-way ANOVA with post hoc multiple comparison using Bonferroni's test. Differences were considered statistically significant when *P* value is less than 0.05 (*P* < 0.05).

## 3. Results

### 3.1. GC-MS Profile of CNME

**Figure 1:**
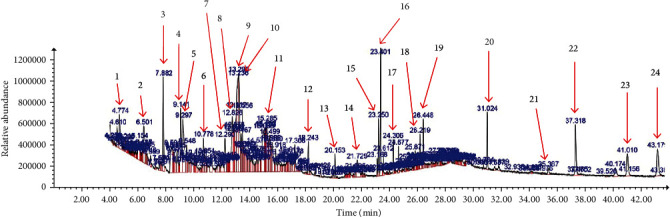
Phytoconstituents from CNME detected using GC-MS with relative time.

The GC-MS profile of CNME is shown in Figure 1. A total of 24 peaks were identified from the CNME extract with the 10 major compounds being (i) lethane, (ii) lethane, (iii) 5-hydroxylmethylfurfural, (iv) 7-azaindole-3-carboaldehyde, (v) phytol, (vi) coumaran/benzofuran, 2,3-dihyro-, (vii) 3-deoxy-d-mannoic acid, (viii) 6-methyluracil, (ix) alpha-tocopherol, and (x) beta-sitosterol (Table 1).

### 3.2. Effect of CNME on Body Weight and FBG Level in Rats

Mean values of the body weights and FBG levels of rats in the five study groups are summarized in Table 2. Two-way ANOVA (group × time (week) as repeated measures) showed significant main effects of time (*P* < 0.0001), group (*P* < 0.0001), and a significant interaction between the two factors (*P* < 0.0.0001) on body weight. Post hoc analysis showed no significant difference in body weight among study groups at week 0. At week 15, the body weights of C and C+CNME groups were significantly increased, whereas it was reduced in the DM group compared to week 0. At week 15, body weights of DM, DM+Met, and DM+CNME groups were significantly lower compared to group C. Body weights of DM+Met and DM+CNME groups were significantly higher compared to those of the DM group at week 15 (Table 2).

FBG levels in the three diabetic groups were significantly higher than those in the nondiabetic control (C) groups. DM+CNME and DM+Met groups showed significantly lower FBG levels compared to the untreated DM group (Table 2). The FBG level of both DM+CNME and DM+Met groups was significantly higher compared to that of the C group.

### 3.3. Vascular Functional Study

#### 3.3.1. Effect of CNME on Endothelium-Dependent and Endothelium-Independent Relaxation

**Figure 2:**
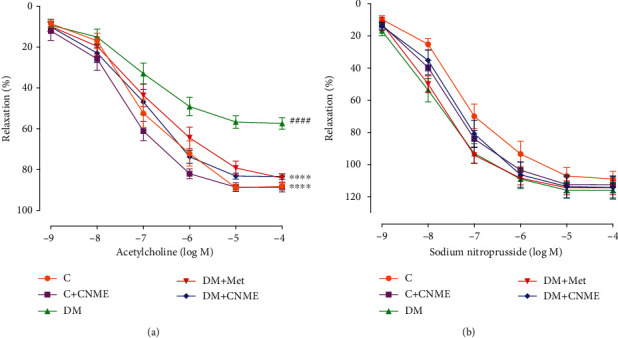
Endothelium-dependent and endothelium-independent relaxations in the thoracic aorta of rats: (a) concentration response curves to ACh (10^−9^ -10^−4^ M); (b) concentration response curves to SNP (10^−9^ -10^−4^ M). Data are presented as mean ± SEM (*n* = 12). Relaxations were expressed as a percentage of the contraction induced by PE (10^−6^ M). ^####^*P* < 0.0001 vs. C. ^∗∗∗∗^*P* < 0.0001 vs. DM.

Endothelium-dependent relaxations to ACh were significantly impaired in the DM group compared to the C group (Figure 2(a), Table 3). No significant difference was seen between the C+CNME group and C group. In addition, no significant differences were seen in DM+CNME and DM+Met groups compared to C groups. Higher *E*_max_ value to ACh in both DM+CNME and DM+Met groups represent higher endothelium-dependent relaxation compared to the DM group (Figure 2(a), Table 3).

SNP-induced endothelium-independent relaxation showed no difference among five groups (Figure 2(b), Table 3).

#### 3.3.2. Effect of CNME on Endothelium-Dependent and Endothelium-Independent Contractions

**Figure 3:**
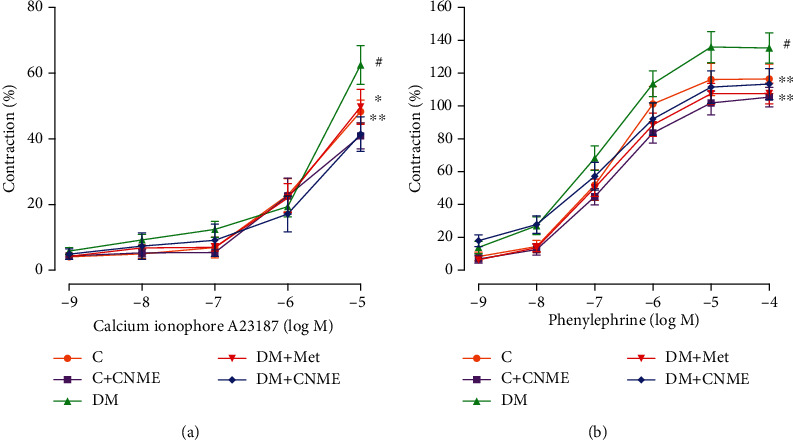
Endothelium-dependent and endothelium-independent contractions in the thoracic aorta of rats: (a) concentration response curves to calcium ionophore A23187 (10^−9^ -10^−5^ M); (b) concentration response curves to PE (10^−9^ -10^−4^ M). Data are presented as mean ± SEM (*n* = 12). Relaxations were expressed as a percentage of the contraction induced by PE (10^−6^ M). ^#^*P* < 0.05 vs. C. ^∗^*P* < 0.05 and ^∗∗^*P* < 0.01 vs. DM.

The DM group showed higher *E*_max_ value in response to calcium ionophore A23187 compared to the C group indicating that T2DM increases the endothelium-dependent contraction (Figure 3(a), Table 4). Endothelium-dependent contractions to calcium ionophore A23187 were significantly lower in DM+CNME and DM+Met groups compared to the DM group (Figure 3(a), Table 4). No significance differences were seen between DM+CNME and DM+Met groups indicating that both of the treatments were comparable. In addition, treatment with CNME in nondiabetic rats does not show any significant difference in endothelium-dependent relaxation and contraction compared to nondiabetic control rats.

The DM group showed higher *E*_max_ value in response to PE compared to C groups indicating that HFD- and low-dose STZ-induced T2DM rat model increases the endothelium-independent contraction (Figure 3(b), Table 4). On the other hand, endothelium-independent contraction to PE in DM+CNME and DM+Met groups was significantly lower compared to that in the untreated DM group (Figure 3(b), Table 4). Treatment with CNME in nondiabetic rats does not showed any significant difference compared to nondiabetic control rats.

### 3.4. Western Blotting

**Figure 4:**
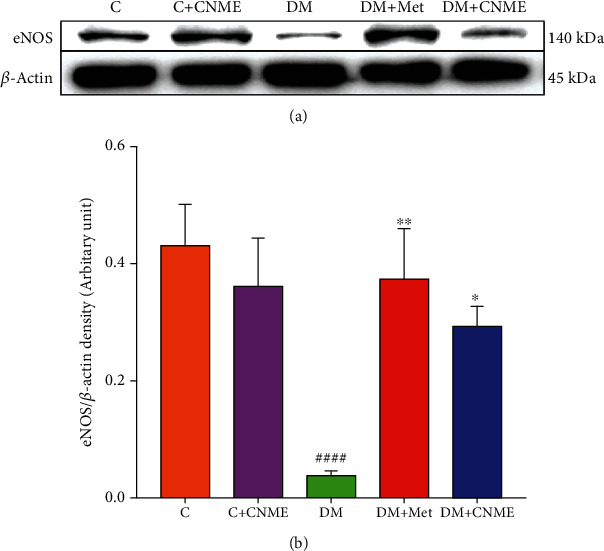
Western blot analysis of eNOS protein expression in the thoracic aorta: (a) representative Western blots showing the expression of eNOS protein of C, C+CNME, DM, DM+Met, and DM+CNME groups; (b) graphical representation of the data, normalized to *β*-actin. Data are presented as mean ± SEM (*n* = 12). ^####^*P* < 0.0001 vs. C. ^∗^*P* < 0.05 and ^∗∗^*P* < 0.01 vs. DM.

The expression of eNOS protein was 10.8-fold lower in the thoracic aorta of the DM group compared to the C group (DM: 0.04 ± 0.01 vs. C: 0.43 ± 0.07 arbitrary units; *P* < 0.0001). The expressions of eNOS protein were 9.3-fold and 7.3-fold higher in the thoracic aorta of both DM+Met (DM+Met: 0.37 ± 0.09 vs. DM: 0.04 ± 0.01 arbitrary units; *P* = 0.0043) and DM+CNME (DM+CNME: 0.29 ± 0.03 vs. DM: 0.04 ± 0.01 arbitrary units; *P* = 0.0476) groups compared to the DM group (Figure 4). No significance difference between DM+CNME and DM+Met groups showed that both groups have comparable effect in increasing eNOS protein expression in the diabetic rats' aorta. The C+CNME group does not show any significant difference compared to the C group.

## 4. Discussion

This study showed that untreated diabetic rats induced by a combination of HFD and low-dose intraperitoneal STZ injection demonstrated higher FBG levels compared to nondiabetic control rats. Untreated diabetic rats also demonstrated impaired endothelium-dependent relaxation and increased endothelium-dependent and endothelium-independent contractions, which indicate ED. Diabetic rats had reduced eNOS protein expression compared to nondiabetic controls. Both DM+CNME and DM+Met groups showed comparable effects in increasing body weight and reducing FBG levels in diabetic rats. Both these groups also increased endothelium-dependent relaxations, reduced endothelium-dependent and endothelium-independent contractions, and increased aortic eNOS protein expression compared to untreated diabetic rats. Treatment of nondiabetic rats with CNME produced no changes in body weight, FBG, endothelial function parameters, and aortic eNOS protein expression compared to nondiabetic control rats.

Untreated diabetic rats in this study demonstrated a reduction in weight compared to nondiabetic controls. The process of gluconeogenesis can be potentiated in diabetes to provide glucose, as the cells of the body do not uptake enough for their metabolism. The substrates for gluconeogenesis are often derived from protein and fat sources, through the processes of glycogenolysis and lipolysis, resulting in loss of tissue protein, muscle wasting, and weight loss [8, 25]. In this study, both diabetic groups treated with CNME or metformin had higher body weight compared to untreated diabetic rats. Cinnamic acid and caffeic acid, phenolic compounds detected in *C. nutans* extract, have been shown to inhibit the process of gluconeogenesis [26]. Attenuation of gluconeogenesis could slow down the breakdown of body fat and proteins thus reducing weight loss associated with diabetes [8].

In this study, HFD and low-dose STZ are used to induce diabetes. High-fat diet feeding can lead to insulin resistance and altered glucose homeostasis [27]. Insulin resistance predisposes to the development of T2DM as the pancreatic *β*-cells start to shut down the production of insulin and no longer create enough insulin to control blood sugar level; this phenomenon leads to hyperglycemia. Compared to the untreated diabetes group, DM+CNME and DM+Met groups had lower FBG levels. The effect of CNME was comparable to metformin in reducing FBG levels in diabetic rats. However, the levels of FBG in DM+CNME and DM+Met groups were still higher compared to those in nondiabetic controls, meaning that CNME and metformin, at the doses used, have hypoglycemic effects, but the levels are not low enough to be in the normal range. Prior in vitro studies [6, 7] have reported that *C. nutans* extracts inhibit *α*-glucosidase enzyme. A more recent study [8] observed that the aqueous extract of *C. nutans* significantly reduced blood glucose concentrations in diabetic rats. Inhibition of *α*-glucosidase enzyme at the small intestine reduces hydrolysing of carbohydrate into glucose, thus reducing its absorption [6, 28]. In the current study, CNME administered to nondiabetic rats did not lower FBG levels indicating that CNME does not produce hypoglycemic effects in the nondiabetic condition.

Diabetic rats in this study had impaired endothelium-dependent relaxation, which is associated with reduced eNOS protein expression. Administration of HFD to diabetic rats increased serum and aortic oxidative stress [29]; it has been reported that oxidative stress is elevated in proportion to the accumulation of advanced glycation end products (AGEs) [30]. AGEs can reduce bioavailability of NO via reducing endothelium eNOS expression [29] and increasing the rate of mRNA degradation [17]. In this study, the DM+CNME group attenuated impaired endothelium-dependent relaxation, which is associated with increased aortic eNOS expression. These effects were comparable with diabetic rats treated with metformin. Improvement in FBG in diabetic rats in this study may reduce AGEs, increasing eNOS protein expression. Reduction in oxidative stress may also lead to improved endothelium-mediated dilatation. *C. nutans* extracts had been shown to show high 2,2-diphenyl-1-picrylhydrazyl (DPPH) radical scavenging activity and increased serum SOD levels in vascular tissues of a hyperlipidemic rat model. Free radicals inactivate NO; thus, reduction in oxidative stress increases vascular NO bioavailability, improving endothelium-mediated relaxation.

In order to evaluate whether or not T2DM alters the function of vascular smooth muscle cells (VSMCs), preparations without endothelium were tested for relaxation with SNP. The responses of smooth muscle cells to SNP were comparable in all groups. This indicates that the impairment of ACh-induced relaxation is not due to changes in the sensitivity of VSMCs to the relaxant effect of NO, showing that seven weeks of diabetes did not affect the smooth muscle cyclic guanosine monophosphate pathway [23].

In this study, calcium (Ca^2+^) ionophore A23187-induced endothelium-dependent contractions were increased in the aorta of untreated diabetic rats. Ca^2+^ ionophore A23187 increases intracellular Ca^2+^ in endothelial cells [31]; this activates the cyclooxygenase (COX) enzyme to generate endothelium-derived contracting factors (EDCF) such as thromboxane A_2_ [32, 33]. ED occurs when there is imbalance between the production of EDCF and EDRF such as NO. In diabetes, augmented vascular oxidative stress increases the production of EDCF thus enhances endothelium-dependent contraction [33, 34]. Our study demonstrated that both DM+CNME and DM+Met groups significantly reduced endothelium-dependent contraction in diabetic rats. This may be due to reduced oxidative stress with improvement in hyperglycemia. This is supported by a study showing that metformin reduces EDCF-mediated contraction by suppressing oxidative stress at vascular endothelial cells [35]. Increased expression of eNOS in diabetic rats treated with CNME or metformin in this study may also contribute to the reduction in endothelium-mediated contraction. Vanhoutte and Tang [33] showed that the production and or/action of EDCF is inhibited by the presence of NO.

Phenylephrine, an *α*-adrenergic receptor agonist stimulates Ca^2+^ entry through voltage-gated and store-operated Ca^2+^ channels and activates Ca^2+^ sensitization pathways such as protein kinase-C (PKC) and Rho-kinase [36]. Activation of PKC increases the myofilament force sensitivity to cytosolic Ca^2+^ concentration and myosin light-chain (MLC) phosphorylation and thereby maintains VSMC contraction with smaller increases in cytosolic Ca^2+^ concentration [37]. In this study, PE-induced endothelium-independent contraction was increased in the thoracic aorta of untreated diabetic rats compared to the nondiabetic controls. In the diabetic state, reduction of NO bioavailability increases Ca^2+^ entry into VSMCs and activated myosin light-chain kinase (MLCK). Activated MCLK phosphorylated the MLC, which leads to crossbridge formation between the myosin heads and the actin filaments resulting in vascular smooth muscle contraction [38]. In our study, diabetics treated with CNME or metformin showed significant reduction in PE-induced endothelium-independent contractions. Metformin activates AMP-activated protein kinase (AMPK) in vascular smooth muscle resulting in inhibition of MLCK activity, thereby diminishing agonist-induced MLC phosphorylation and contractile response [39, 40]. Similar to metformin, protocatechuic acid, a phenolic compound present in *C. nutans* extract [4], has also been shown to activate AMPK in VSMCs of rat thoracic aorta, which prevents VSMC proliferation and atherogenesis [41].

As shown by the GCMS data, a number of bioactive compounds were detected in CNME which include coumaran/benzofuran,2,3-dihyro-, 5-hydroxylmethylfurfural (5-HMF), 7-azaindole-3-carboaldehyde, 3-deoxy-d-mannoic acid, lethane, phytol, alpha-tocopherol (vitamin E), beta-sitosterol, 6-methyluracil, and 4H-pyran-4-one,2-3-dihydro-3,5-dihydroxy-6-methyl. In this study, the hypoglycemic and vascular protective effects of CNME could be due to the presence of bioactive compounds such as 5-HMF, squalene, beta-sitosterol, and stigmasterol [42–44]. 5-HMF is a cyclic aldehyde that possesses antioxidant, anti-inflammatory, and hypoglycemic activities [43]. In addition, 5-HMF exhibits a cardioprotective effect in ischemia/reperfusion injury that is mediated by inhibition of L-type Ca^2+^ channels in isolated normoxic perfused heart of rats [45]. Squalene is a triterpene that acts as a natural antioxidant [44, 46]. *β*-Sitosterol and stigmasterol had previously been reported to have antidiabetic and anti-inflammatory properties [42, 47]. Other than that, phytol, 4H-pyran-4-one,2-3-dihydro-3,5-dihydroxy-6-methyl, and neophytadiene possess antioxidant properties [44, 48]. Thus, methanolic extract of *C. nutans* leaves could be a potential candidate to be explored further for use as an adjunct to other hypoglycemic agents, with concurrent beneficial effects on diabetic vasculopathy.

## 5. Conclusion

Findings from this study showed that CNME-augmented endothelium-dependent relaxation reduced endothelium-dependent and endothelium-independent contraction in the aorta of diabetic rats. This likely occurs due to increased expression of vascular eNOS protein, which increases the bioavailability of NO. In addition, treatment with CNME reduced the level of FBG in diabetic rats, although at the dosage used, it did not reach the normal range. In addition, antioxidant and hypoglycemic properties from bioactive compound(s) present in CNME may reduce oxidative stress and potentiate to improve NO bioavailability. The effects of CNME in lowering blood sugar levels, improving endothelial dysfunction, and increasing eNOS expression are comparable to metformin, a widely used antidiabetic drug.

## Figures and Tables

**Table 1:** Phytoconstituents identified in the CNME by GC-MS.

Peak	Name of the compound	Peak area (%)
1	1,3-Propanediamine, N,N-dimethyl	1.47
2	6-Methyluracil	1.96
3	4H-Pyran-4-one, 2-3-dihydro-3,5-dihydroxy-6-methyl	2.20
4	Coumaran/benzofuran,2,3-dihyro-	2.31
5	5-Hydroxylmethylfurfural	3.10
6	3-Methoxymethcathinone	1.09
7	Pyridine, 2,5-dimethyl-	1.43
8	7-Azaindole-3-carboaldehyde	2.93
9	Lethane	4.92
10	Lethane	4.34
11	3-Deoxy-d-mannoic acid	2.13
12	Neophytadiene	0.76
13	Hexadecanoic acid, methyl ester	0.56
14	9H-Pyridol[3,4-]indole, b-carboline	1.20
15	9,12,15-Octadecatrienoic acid, methyl ester	1.02
16	Phytol	2.65
17	Hexadecanamide	0.61
18	9-Octadecenamide	0.71
19	Octadecanamide	0.78
20	Squalene	1.18
21	Beta-tocopherol, vitamin E	0.26
22	Alpha-tocopherol, vitamin E	1.91
23	Stigmasterol	1.20
24	Beta-sitosterol	1.57

**Table 2:** Body weight and FBG changes in nondiabetic and HFD-fed diabetic rats.

	C	C+CNME	DM	DM+Met	DM+CNME
Initial body weight (g) (week 0)	308.30 ± 10.57	292.50 ± 14.16	319.60 ± 10.13	313.80 ± 12.82	307.20 ± 9.54
Final body weight (g) (after treatment, week 15^th^)	409.30 ± 17.12^$$$$^	409.90 ± 16.33^$$$$^	260.50 ± 7.09^$,####^	321.60 ± 12.53^###,^^∗^	330.90 ± 13.69^##,^^∗^
FBG (mmol/L) (after treatment)	4.82 ± 0.20	4.78 ± 0.29	28.16 ± 0.85^####^	18.33 ± 0.91^####,^^∗∗∗∗^	19.52 ± 0.54^####,^^∗∗∗∗^

Data are presented as mean ± SEM (*n* = 12). ^$^*P* < 0.05 and ^$$$$^*P* < 0.0001, final body weight versus initial body weight within the same group. ^##^*P* < 0.01, ^###^*P* < 0.001, and ^####^*P* < 0.0001 vs. C. ^∗^*P* < 0.05 and ^∗∗∗∗^*P* < 0.0001 vs. DM.

**Table 3:** Relaxations to ACh and SNP in thoracic aortas.

	C	C+CNME	DM	DM+Met	DM+CNME
Acetylcholine (ACh)					
*E*_max_ (%)	90.14 ± 1.37	88.74 ± 2.13	57.82 ± 2.62^####^	84.06 ± 1.98^∗∗∗∗^	83.87 ± 1.27^∗∗∗∗^
Sodium nitroprusside (SNP)					
*E*_max_ (%)	108.90 ± 4.63	112.40 ± 4.99	124.00 ± 8.30	114.40 ± 4.18	115.40 ± 6.89

Data are presented as mean ± SEM (*n* = 12). ^####^*P* < 0.0001 vs. C. ^∗∗∗∗^*P* < 0.0001 vs. DM.

**Table 4:** Contractions to calcium ionophore A23187 and PE in thoracic aortas.

	C	C+CNME	DM	DM+Met	DM+CNME
Calcium ionophore A23187					
*E*_max_ (%)	48.30 ± 3.54	44.14 ± 4.82	69.61 ± 5.55^#^	44.54 ± 4.94^∗^	41.63 ± 5.11^∗∗^
Phenylephrine (PE)					
*E*_max_ (%)	105.60 ± 6.47	103.50 ± 7.21	140.00 ± 9.07^#^	101.80 ± 6.06^∗∗^	98.06 ± 6.73^∗∗^

Data are presented as mean ± SEM (*n* = 12). ^#^*P* < 0.05 vs. C. ^∗^*P* < 0.05 and ^∗∗^*P* < 0.01 vs. DM.

## Data Availability

Data is available on request.
